# 3D Molecular Imaging of Stratum Corneum by Mass Spectrometry Suggests Distinct Distribution of Cholesteryl Esters Compared to Other Skin Lipids

**DOI:** 10.3390/ijms232213799

**Published:** 2022-11-09

**Authors:** Peter Sjövall, Sebastien Gregoire, William Wargniez, Lisa Skedung, Gustavo S. Luengo

**Affiliations:** 1 RISE Research Institutes of Sweden, Materials and Production, SE-50115 Borås, Sweden; 2 L’Oréal Research and Innovation, 93601 Aulnay-sous-Bois, France; 3 RISE Research Institutes of Sweden, Bioeconomy and Health, SE-11428 Stockholm, Sweden

**Keywords:** 3D ToF-SIMS, stratum corneum, layer structure, lipid distribution, cholesteryl esters

## Abstract

The crucial barrier properties of the stratum corneum (SC) depend critically on the design and integrity of its layered molecular structure. However, analysis methods capable of spatially resolved molecular characterization of the SC are scarce and fraught with severe limitations, e.g., regarding molecular specificity or spatial resolution. Here, we used 3D time-of-flight secondary ion mass spectrometry to characterize the spatial distribution of skin lipids in corneocyte multilayer squams obtained by tape stripping. Depth profiles of specific skin lipids display an oscillatory behavior that is consistent with successive monitoring of individual lipid and corneocyte layers of the SC structure. Whereas the most common skin lipids, i.e., ceramides, C24:0 and C26:0 fatty acids and cholesteryl sulfate, are similarly organized, a distinct 3D distribution was observed for cholesteryl oleate, suggesting a different localization of cholesteryl esters compared to the lipid matrix separating the corneocyte layers. The possibility to monitor the composition and spatial distribution of endogenous lipids as well as active drug and cosmetic substances in individual lipid and corneocyte layers has the potential to provide important contributions to the basic understanding of barrier function and penetration in the SC.

## 1. Introduction

The molecular architecture of the outermost skin layer, the stratum corneum (SC), is critical for the function of the skin as a chemical barrier between our interior bodies and the external environment [1,2,3]. The overall structure of the SC is often described in terms of a “brick-and-mortar” model, in which layers of flat, protein-rich, non-viable cells called corneocytes are embedded in a lipid matrix. Whereas this model provides a simple yet representative outline of the SC structure, a closer look reveals a rich complexity, both in terms of molecular composition and spatial distributions [3,4,5,6,7]. For example, the lipid phase of the SC has a unique composition (compared to typical viable cells) with ceramides, cholesterol and long-chain fatty acids (C24:0 and C26:0) as the main components, which are organized in well-ordered multilayer structures in the space that separates the flat corneocyte layers. The corneocytes are mainly made up of keratin fibers with an outer shell of cross-linked proteins, called the cornified envelope, onto which a lipid layer composed of ceramides is covalently bound to ensure tight binding to the inter-corneocyte lipid layers. The lamellar lipid layer structure between corneocytes is considered to be of particular importance for the chemical barrier properties, and several skin disorders have been associated with distortions in the composition and/or structural organization of the lipid phase [3,5,6,8,9]. A clear understanding of the barrier properties of SC is essential in dermatology and cosmetics because the SC is the substrate that all topical ingredients encounter upon application. Depending on its chemical properties, the ingredient is expected to follow different penetration pathways across the SC, either through the corneocytes, via the lipid matrix, or through appendages, such as hair follicles. In all cases, it is of essential importance to control the final location of an active ingredient, in order to ensure safety and performance. Finally, optimal target localization of active ingredients requires detailed and accurate representations of the stratum corneum structure [10,11,12], and novel, more evolved models need to incorporate accurate chemical descriptions of, e.g., penetration and inter cell adhesion processes. These factors have recently stimulated the development of multiple methods to image and study skin in vitro or in vivo [13,14]. 

Detailed molecular and structural information about SC architecture has been obtained using a variety of powerful methods, including mass spectrometry for lipid composition [15,16,17] and transmission electron microscopy and X-ray diffraction methods for structural evaluation [1,18]. FTIR and Raman imaging are powerful methods for determination of spatial/molecular distributions in the skin [19,20,21]. Here, the molecular information is provided by vibrational signals that can be associated to different types of molecular species. However, identification and mapping of specific molecular species can only be made after isotopic labelling.

Imaging mass spectrometry (IMS) is a promising approach for analysis of biological tissues, as it provides simultaneous identification and spatial mapping of specific molecular species in the sample [22]. Imaging matrix-assisted laser desorption ionization (MALDI) MS is capable of imaging molecular species with high specificity and sensitivity [23] and the technique has recently been used successfully to quantify drug concentrations in skin cross sections [23,24,25]. However, the typical spatial resolution of imaging MALDI does not allow for interrogation of different components of SC [24].

Time-of-flight secondary ion mass spectrometry (ToF-SIMS) is an IMS method that is capable of label-free 2D imaging of specific molecules at spatial resolutions down to the sub-micrometer range [26,27,28]. In skin samples, recent studies have demonstrated mapping of endogenous and exogenous compounds (cosmetics, drugs, metal ions) at high spatial resolution using ToF-SIMS [29,30,31,32,33,34,35,36,37,38,39,40,41]. Furthermore, 3D molecular mapping of organic materials can be achieved by repeated 2D mapping in parallel with gradual removal of material from the sample surface by sputtering using large Ar cluster ions (e.g., Ar_2000_^+^). This 3D ToF-SIMS analysis takes advantage of the extreme surface specificity of ToF-SIMS (1–10 nm) and the minimal molecular damage caused by the Ar cluster ions [42,43,44]. The capabilities of 3D ToF-SIMS to provide molecular 3D information at high spatial resolution have been demonstrated for different types of samples, including cells and tissues [45,46,47] and organic reference samples [44]. The method has also been applied to skin, where 3D permeation profiles of ascorbic acid (vitamin C) and precursor molecules were recorded and the effect of a permeation enhancing formulation was investigated [40]. In addition, the recently developed 3D OrbiSIMS technique, which combines 3D ToF-SIMS with Orbitrap mass spectrometry, was shown to provide detailed depth profile information about a large range of specific molecular skin components from the skin surface, through the SC and into the viable epidermis [48].

In the present study, we explored the possibility to monitor the layered molecular architecture of stratum corneum by 3D ToF-SIMS analysis of corneocyte multilayer fragments sampled by tape stripping. We showed that the layer structure of the SC is clearly visible in the data and that the molecular composition of individual lipid and corneocyte layers can be characterized. Furthermore, cholesteryl oleate was identified and shown to have a 3D distribution that is distinct from the distributions of the major skin lipids, i.e., ceramides, free cholesterol and C24:0 and C26:0 fatty acids, thereby suggesting a different function of cholesteryl esters in the stratum corneum structure.

## 2. Results

**Figure 1:**
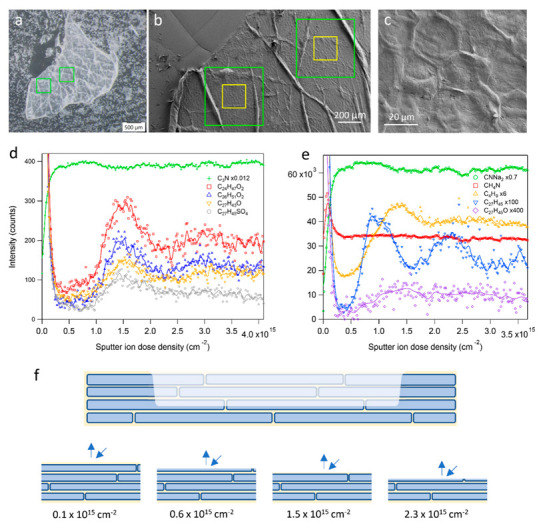
3D ToF-SIMS analysis of SC multilayer fragment. (**a**) Light microscopy and (**b**,**c**) SEM images of the SC fragment deposited on the tape surface after tape stripping. Squares in (**b**) indicate sputter and analysis areas (green and yellow, respectively) for the 3D ToF-SIMS analyses presented here. The magnified image in (**c**) is from inside the left analysis area in (**b**). (**d**) Depth profiles of negative ions representing proteins (C_3_N^−^), C24:0 and C26:0 fatty acids (C_24_H_47_O_2_^−^ and C_26_H_51_O_2_^−^, respectively), cholesterol (C_27_H_43_O^−^) and cholesterol sulfate (C_27_H_45_SO_4_^−^). (**e**) Depth profiles of positive ions representing proteins (CNNa^+^ and CH_4_N^+^, respectively), lipid fragment (C_4_H_9_^+^), and cholesterol-related ions (C_27_H_45_^+^ and C_27_H_45_O^+^, respectively). Symbols are acquired data points and lines are results after digital smoothing of the data. Some data were multiplied by factors (as indicated) to improve visibility. The horizontal axis corresponds to sample depth, here represented by the applied sputter ion dose density. (**f**) Schematic representation of the 3D analysis of the corneocyte multilayer sample. Successive removal of material from the sample surface exposes alternately the lipid phase and the corneocyte interior, which were monitored separately due to the strong surface sensitivity of ToF-SIMS.

Multilayer corneocyte fragments of stratum corneum (SC) were analyzed by 3D ToF-SIMS with the aim of monitoring the 3D distribution of skin lipids in the layered structure (see Table 1 for peaks/ions used to represent different molecular components). Since the SC fragments were removed from the skin sample by tape stripping, the surface of the multilayer fragment represents an interface between deeper corneocyte layers, whereas the corneocyte layer closest to the original skin surface is attached to the tape at the bottom of the SC fragment. Light microscopy and SEM images of the analyzed SC fragment showed an overall flat surface with a pattern of wrinkles crossing the otherwise flat regions (Figure 1a,b). Magnified images showed characteristic corneocyte structures (Figure 1c), thus confirming that the surface of the SC fragment represents the interface between two corneocyte layers in the SC structure. The thickness of the SC fragment was not explicitly measured, but SEM images of the fragment edges indicated that the fragment was made up of at least 8–10 corneocyte layers (Supplementary Figure S1).

Depth profiles from 3D ToF-SIMS analysis of the SC fragment showed oscillatory features that reflected the layer structure of SC (Figure 1d,e). Starting from the surface (0.0 cm^−2^ sputter ion dose density), the signal intensity of the skin lipids, in Figure 1d represented by C24:0, C26:0, cholesterol and cholesterol sulfate, was very high but decreased rapidly during the initial sputtering, whereas the signal of ions representing proteins increased to a level that was largely constant during the remainder of the depth profile. These observations are consistent with the presence of a thin layer of skin lipids on top of the corneocyte layer on the SC fragment surface. The initial decrease was followed by a depth interval (0.3–0.8 × 10^15^ cm^−2^) with very low signal intensities of the skin lipids, indicating that this interval represents sputter removal of the topmost corneocyte layer. However, at around 1 × 10^15^ cm^−2^, the skin lipid intensities started to increase in a concerted manner to all reach a maximum at around 1.5 × 10^15^ cm^−2^, after which they again decreased. The oscillatory behavior of the skin lipid intensities continued with a minimum and maximum at around 2.3 × 10^15^ and 3 × 10^15^ cm^−2^, respectively, which, however, were considerably less pronounced than the first oscillatory features. These features are consistent with sputtering through successive corneocyte layers, where the skin lipid maxima at 1.5 × 10^15^ and 3 × 10^15^ cm^−2^ represented lipid matrix layers below the first and second corneocyte layers, respectively (see Figure 1f). The relatively broad features and low signal intensities (compared to the skin lipid intensities on the top surface) of the interior lipid matrix layers were likely caused by 2D inhomogeneities in the corneocyte thickness (e.g., associated to the keratin fibers that make up the corneocyte interior structure) and the very thin nature of the lipid layers between corneocytes, resulting in an only partial and gradually changing exposure of the lipid layer over an extended depth interval.

**Figure 2:**
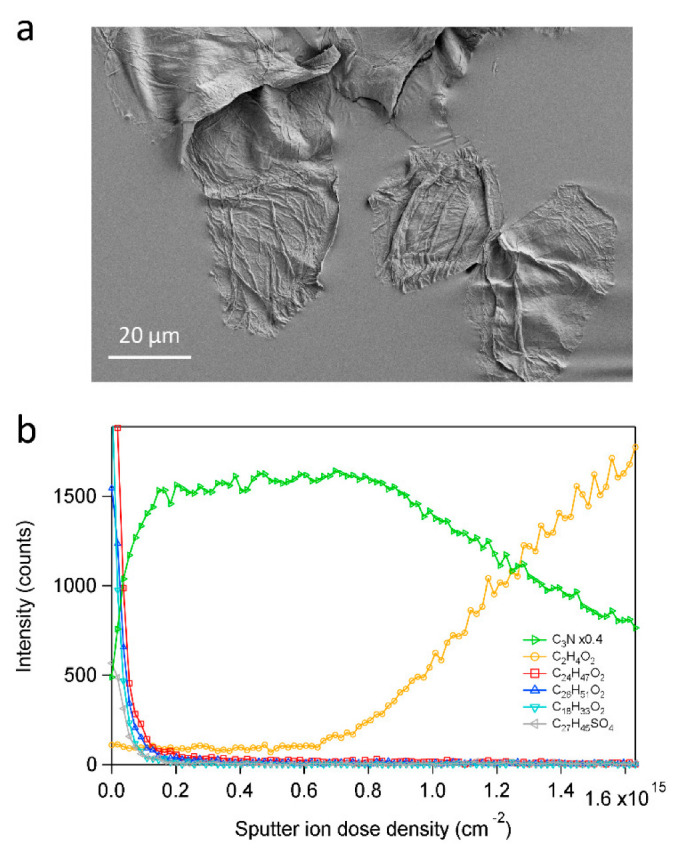
3D ToF-SIMS analysis of single corneocyte layer. (**a**) SEM image and (**b**) negative ion depth profiles of single corneocyte layers deposited on tape substrate by tape stripping. The monitored ions represent proteins (C_3_N^−^), tape (C_2_H_4_O_2_^−^), C24:0 and C26:0 fatty acids (C_24_H_47_O_2_^−^ and C_26_H_51_O_2_^−^, respectively), oleic acid (C18:1, C_18_H_33_O_2_^−^) and cholesterol sulfate (C_27_H_45_SO_4_^−^). The imaged area in (**a**) was not analyzed by 3D ToF-SIMS.

The interpretation of the oscillatory features in Figure 1d as separate corneocyte layers was verified by depth profiles acquired for single corneocyte layers on the tape strip sample (Figure 2). These depth profiles showed initially decreasing signal intensities for the skin lipids, indicating the presence of a top lipid layer, similar to the SC fragment surface. More importantly, however, is the observation that the protein fragment ion signal intensity, after an initial increase, reached a value that was approximately constant up to a sputter ion dose density of about 0.8 × 10^15^ cm^−2^, after which it gradually decreased. In parallel to the decreasing protein signal, the signal intensity of a fragment ion representing the underlying tape surface started to increase, indicating the gradual removal of the attached corneocytes and increasing exposure of the underlying tape surface. Interestingly, the signal intensity of the underlying tape surface started to increase at approximately the same sputter ion dose density as the skin lipid intensity started to increase for the SC multilayer sample. This shows that the position of the skin lipid maxima in Figure 1d is consistent with the sputter ion dose density required to remove one corneocyte layer, as determined from Figure 2b.

Oscillatory features, similar to those observed for the (negative) skin lipid ions, were observed for positive ions (Figure 1e, separate measurement). Positive fragment ions representing proteins and lipids showed similar depth profiles as the corresponding negative ions, but with less specificity than the negative skin lipid ions. For example, the CH_4_N^+^ ion was generated by proteins (i.e., in the corneocytes), but also by ceramides in the lipid matrix, resulting in a constant signal intensity for most part of the depth profile, but also a pronounced peak at the initial (top) lipid layer. The C_4_H_9_^+^ signal intensity mainly represented lipids, but also contained contributions from other organic molecules.

A particularly interesting observation from Figure 1e is the pronounced oscillatory behavior in the depth profile of the C_27_H_45_^+^ ion, which corresponds to dehydroxylated cholesterol and is often used to monitor cholesterol by ToF-SIMS in tissue samples [42,49]. Interestingly, compared to the skin lipid depth profiles in Figure 1d, the oscillatory pattern in the C_27_H_45_^+^ depth profile was significantly shifted to lower sputter ion dose densities; whereas the skin lipid profiles have maxima at around 1.5 × 10^15^ cm^−2^, the first maximum in the C_27_H_45_+ profile occurred at 0.9–1.0 × 10^15^ cm^−2^, i.e., significantly closer to the original surface than the maxima for the skin lipids. Furthermore, the depth profile of the molecular cholesterol ion (C_27_H_45_O^+^), although at a considerably higher noise level, showed a maximum at 1.5 × 10^15^ cm^−2^, i.e., similar to the negative ion skin lipids, including the negative molecular cholesterol ion (C_27_H_43_O^−^, Figure 1d).

**Figure 3:**
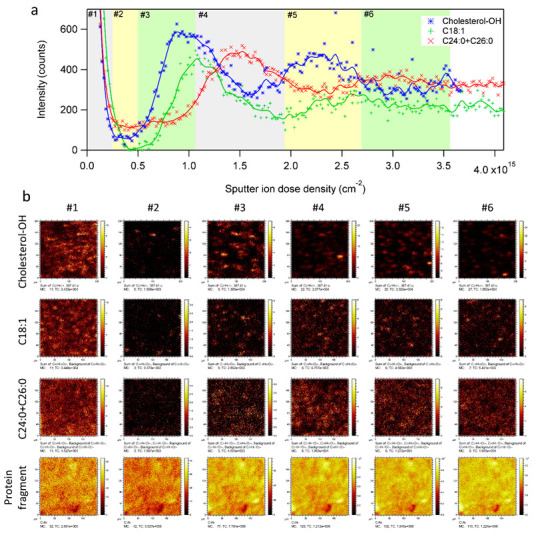
3D ToF-SIMS analysis of SC multilayer fragment. (**a**) Depth profiles of dehydroxylated cholesterol (C_27_H_45_^+^, positive ions), oleic acid (C_18_H_33_O_2_^−^, negative ions) and C24:0 + C26:0 fatty acids (added signal from C_24_H_47_O_2_^−^ and C_26_H_51_O_2_^−^, negative ions). (**b**) 2D ion images generated from increasing depths in the 3D analysis volume (depth intervals indicated in (**a**)) of the lipid ions in (**a**) and of a protein fragment ion (C_3_N^−^, negative ions). Note the highly inhomogeneous and changing distribution of dehydroxylated cholesterol and C18:1 fatty acid ions with increasing depth into the skin sample. Positive and negative ion data were acquired in separate measurements.

The contradictory observations for the different cholesterol ions can be resolved if we consider the possibility that the different cholesterol-related ions may represent different forms of cholesterol in the sample. In a previous study [50], it was found that the signal intensity ratio of C_27_H_45_O^+^ and C_27_H_45_^+^ was significantly higher for free cholesterol than for cholesterol linoleate (see Supplementary Figure S2), indicating that the signal intensity of C_27_H_45_^+^ contained a larger relative contribution from cholesterol esters, whereas that of C_27_H_45_O^+^ mainly represented free cholesterol. Support for the possibility that the dehydroxylated cholesterol ion (C_27_H_45_^+^) mainly represents cholesterol esters was provided by the depth profile of the C18:1 fatty acid (oleic acid, C_18_H_33_O_2_^−^), which displayed a similar shift relative to those of the other skin lipids (Figure 3a), thus suggesting that the shifted depth profiles represent the presence of cholesteryl oleate in the sample. Thus, the shifted depth profiles of the oleate ion and the dehydroxylated cholesterol ion relative to the other skin lipids indicate the presence of cholesteryl oleate in the sample, and that this compound has a different spatial distribution in the SC structure, as compared the other skin lipids (including free cholesterol).

The 3D distribution of skin lipids in the SC layer structure is shown in Figure 3b as 2D ion images extracted from different depths in the 3D analysis volume (the depth intervals are indicated in Figure 3a by different background colors). Thus, the first column of images (#1) shows the lateral distributions of selected ions in the lipid-rich top region of the SC fragment sample. The following columns show images of the same ions in the (#2) protein-rich and lipid-deficient region corresponding to corneocytes, (#3) the following cholesteryl oleate-rich region, (#4) the region containing the first maximum of the other skin lipids, (#5) second cholesteryl oleate-rich region and (#6) second region rich in the other skin lipids. Whereas the 2D distribution of the protein fragment ion was relatively unchanged throughout the depth of the analysis volume, the images of the dehydroxylated cholesterol and C18:1 fatty acid ions were highly inhomogeneous, with spot-like features that changed considerably during the course of the depth profile (note that the dehydroxylated cholesterol and C18:1 images were acquired in different measurements and thus monitor different positions of the SC sample). In contrast, the 2D distribution of C24:0 + C26:0 was considerably more homogeneous. These observations indicate a spot-like distribution of cholesteryl oleate in the SC structure, separate from a more homogeneous distribution of C24:0 and C26:0 fatty acids, both with regards to the 2D distribution parallel to the surface and in the depth direction.

**Figure 4:**
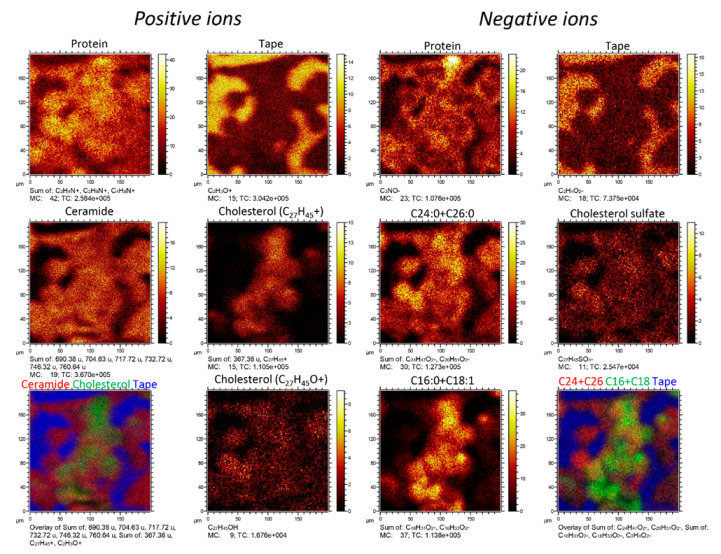
ToF-SIMS ion images of a single corneocyte layer on tape substrate prior to argon cluster sputtering. Positive and negative ion data were acquired in separate measurements but from the same sample position.

The deviating spatial distribution of cholesteryl esters compared to the other skin lipids is further supported by ion images obtained from the surface of a single corneocyte layer on a tape substrate (Figure 4). These images displayed a relatively homogeneous distribution of the major skin lipids (ceramides, C24:0 and C26:0 fatty acids, cholesterol, cholesterol sulfate) on the surface of the attached corneocytes. However, the ion images of dehydroxylated cholesterol and C16:0 + C18:1 fatty acids revealed 2D distributions that were very similar between themselves, but distinctly different from those of the major skin lipids, displaying a more inhomogeneous distribution that does not cover the entire corneocyte surface.

**Figure 5:**
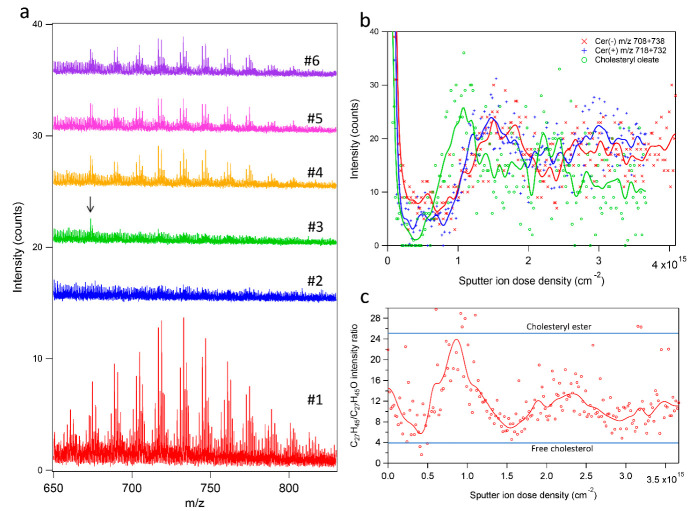
Variation in ceramide and cholesteryl oleate signal intensities with increasing sample depth. (**a**) Positive ion ToF-SIMS spectra in a mass range including molecular ceramide ions extracted from different depth intervals as shown in Figure 3a. The signal intensity of each spectrum has been normalized with respect to the different acquisition times of each depth interval and an offset has been applied for each spectrum for improved presentation. The arrow indicates the peak assigned to cholesteryl oleate. (**b**) Depth profiles of selected ceramide ions in positive and negative ion mode (separate measurements) and for the molecular ion of cholesteryl oleate (C_45_H_78_O_2_Na^+^, positive ions). (**c**) Variation of the C_27_H_45_^+^/C_27_H_45_O^+^ signal intensity ratio with increasing depth into the skin sample. Note clear oscillations indicating changes between cholesterol esters and free cholesterol with increasing depth. Symbols in (**b**,**c**) are data point and solid lines are results of digital smoothing of the data.

Mass spectra from different depth intervals in the 3D analysis volume provided information about the lipid composition in specific layers of the SC structure, as shown in Figure 5a for a mass region including peaks corresponding to molecular ceramide ions. The spectra were extracted from the same depth intervals as the ion images in Figure 3b (indicated in Figure 3a). The spectra were normalized with respect to acquisition time, which means that the signal intensities can be compared to estimate the corresponding lipid concentrations in the different layers. The spectrum from the top lipid layer (#1) revealed a range of peaks that could be identified as sodium-cationized molecular ions of ceramides with known abundances in SC (see ref [39] for specific assignments). As expected, the signal intensities of the ceramide-related peaks were reduced to the noise level in the corneocyte layer (#2) but appeared again in the following lipid layer (#4). The ceramide peaks were absent in the preceding layer (#3), which included the cholesterol ester maximum in the depth profiles. However, the spectrum of this layer (#3) contained a peak at *m*/*z* 673.66 (arrow in Figure 5a) that was identified as the sodium-cationized molecular ion of cholesteryl oleate (C_45_H_78_O_2_Na^+^), and this peak remained in the spectra of the deeper layers (#4–#6).

The identification of the peak at *m*/*z* 673.66 as cholesteryl oleate was verified by the depth profile of this peak in Figure 5b, displayed together with depth profiles of peaks corresponding to molecular ceramide ions in positive and negative 3D ToF-SIMS data. Despite considerable noise in the data, the cholesteryl oleate depth profile showed a distinct maximum at a sputter ion dose density of approximately 1.0 × 10^15^ cm^−2^, clearly shifted from coinciding maxima of the ceramide depth profiles at around 1.5 × 10^15^ cm^−2^, in good agreement with the depth profiles related to cholesteryl esters and major skin lipids, respectively, in Figure 1d,e and Figure 3a. These depth profiles thus confirm the deviating spatial distribution of cholesteryl oleate compared to the major skin lipids and the identification of the peaks at *m*/*z* 673.66 as cholesteryl oleate molecular ion.

As discussed above, the presence of cholesterol as cholesteryl ester or free cholesterol in the skin tissue can be assessed using the signal intensity ratio of the C_27_H_45_^+^ and C_27_H_45_O^+^ ions. Figure 5c displays this signal intensity ratio during the course of the 3D ToF-SIMS analysis of the SC sample, where the horizontal lines indicate the ratios determined for free cholesterol and cholesteryl linoleate, respectively [50]. Interestingly, the diagram reveals an oscillatory behavior indicating that the form of cholesterol changes in the SC structure, occurring mainly as free cholesterol at the center of the lipid layers, 1.5 × 10^15^ cm^−2^ and 3.0 × 10^15^ cm^−2^, but mainly as cholesteryl esters in adjacent layers.

## 3. Discussion

The results in this work demonstrate that the 3D distributions of specific lipids in stratum corneum can be determined by 3D ToF-SIMS. Oscillations in lipid depth profiles correlated well with successive removal of single corneocyte layers and monitoring of the lipid composition in the intermediate lipid layers, similarly to what has previously been observed for hair [51]. Furthermore, 2D ion images at different depth levels provided information about the 2D distributions of specific lipids at varying depth levels of the stratum corneum structure. The capacity to monitor the 3D distribution of specific lipids was demonstrated by results showing an inhomogeneous 3D spatial distribution of cholesterol oleate, clearly deviating from the more homogeneous distributions observed for the other skin lipids in the lipid layers of the SC structure, including ceramides, C24:0 and C26 fatty acids, cholesteryl sulfate and free cholesterol.

A particularly interesting aspect of the results is that they demonstrate the capability to separately characterize the molecular composition of the corneocyte layers and the lipid matrix, thus opening up for the possibility to study the partitioning of exogeneous compounds between corneocytes versus lipid matrix as they penetrate the SC. Such measurements would potentially provide important information about the penetration mechanism for different exogenous molecules as well as the effect of penetration enhancers.

Whereas the composition and spatial organization of the main skin lipids in the SC lipid matrix have previously been characterized in detail, much less information is available for cholesteryl esters in the skin. The concentration of cholesteryl esters in SC has been reported to be around 10% of the total lipid content in human SC [6,52], but data on their spatial distribution has not, to our knowledge, been reported. In a study of skin lipid model films, it was found that cholesteryl oleate does not mix with other skin lipid components and it was suggested that cholesteryl esters extracted from stratum corneum may largely represent contaminants [53]. Whereas the observations in the present work are consistent with the non-mixing properties of cholesteryl oleate, they are clearly inconsistent with a contamination origin. In contrast, our results provide strong evidence for the presence of cholesteryl esters inside stratum corneum and, instead, indicate an alternative function of cholesteryl esters in stratum corneum than the other major skin lipids. Indeed, cholesteryl esters are more hydrophobic than free cholesterol and occur in the human body mainly in lipoprotein particles or lipid droplets, to allow for, e.g., cholesterol transport and storage. Transformation between free cholesterol and cholesteryl esters is controlled by complex enzymatic mechanisms, which are believed to be involved in variable diseases such as Alzheimer’s disease [54] and atherosclerosis [55,56,57]. Our direct observations of an inhomogeneous distribution of cholesteryl esters in stratum corneum are thus consistent with the hydrophobic properties and particle-like distributions of cholesteryl esters in other tissues.

Our results demonstrate the capacity to characterize the molecular composition of the lipid layers on top and below the first corneocyte layers. However, the oscillations were less pronounced for the deeper layers, indicating that mixing between layers becomes increasingly extensive for the deeper layers. The reason for this mixing is most likely the inhomogeneous structure of the corneocytes, which results in varying sputter dose densities required to access the underlying lipid layer for different positions within the analysis area. Although this increased mixing limits the possibility to probe multiple lipid layers in a single measurement, access to lipid layers at different depths of SC may be obtained by successive removal of single corneocyte layers by tape stripping prior to analysis.

## 4. Materials and Methods

### 4.1. Skin Samples and Preparation

Ex vivo full thickness frozen human skin obtained from abdominal plastic surgery was used (mean thickness measured with caliper: 2.80 ± 0.40 mm). Ten successive tape strip samples were prepared from each skin sample using D-squame disks D100 (CuDerm Corporation, Dallas, TX, USA) with a diameter of 22 mm. Each individual strip was placed in a Petri dish, then shipped on dry ice and stored at −20 °C until analysis (approximately 2 weeks).

The large majority of tape strip samples comprised patches of corneocytes transferred from the skin sample to the tape solely as single corneocyte layers, as expected from the normal preparation of tape strip samples [58]. However, it was observed by light microscopy (and later by electron microscopy) that a few tape strip samples contained patches of multiple corneocyte layers that had been transferred to the tape. Evidently, multiple corneocyte layers may remain intact during tape stripping due to strong cohesion forces between the individual layers, as studied previously [59]. The focus of this study is data acquired from such patches of corneocyte multilayers. 

The 3D-ToF-SIMS data presented in Figure 1, Figure 2, Figure 3 and Figure 5 were acquired on a corneocyte multilayer patch from an untreated skin sample (female, 39 years old, Caucasian, abdominal skin). Similar 3D ToF-SIMS results (and data for Figure 4) were obtained from another skin sample (same donor) treated with a simple mixture of Water/Dipropylene Glycol (50/50 *w*/*w*, 500 µL cm^−2^) on a Franz cell filled with PBS buffer for 4 h. The skin temperature was controlled at 32 ± 1 °C and the skin integrity was checked with trans-epidermal water loss (TEWL). TEWL values were below a defined limit based on historical data. No skin samples were rejected. After incubation in the Franz cell and prior to preparation of the tape strip samples, the skin surface was washed with a cotton tip soaked with gentle detergent solution at 10% in water, then rinsed with a second cotton tip soaked in water and dried with at least a third cotton tip.

### 4.2. Time-of-Flight Secondary Ion Mass Spectrometry (ToF-SIMS)

In ToF-SIMS, spatially resolved molecular information is obtained by bombarding the sample surface with high energy (primary) ions and acquiring mass spectra of the (secondary) ions that are emitted into vacuum during the collision process. By scanning the focused beam of primary ions over a selected analysis area of the sample, individual mass spectra are obtained from each pixel and the acquired data can then be presented as ion images, showing the lateral distribution of specific ions, or mass spectra from selected regions of interest (ROIs) within the analysis area. In 3D ToF-SIMS, three-dimensional mass spectrometry data is obtained by parallel 2D ToF-SIMS analysis and erosion of material from the sample surface by ion sputtering. In the present work, the analysis was carried out by repeated cycles of 2D ToF-SIMS analysis using a focused Bi_3_^+^ primary ion beam, and ion sputtering with Ar_2000_^+^ ions from a gas cluster ion source, thus producing a stack of 2D mass spectrometry data from increasing depths, starting from the original surface of the sample. The results can then be presented as (i) depth profiles showing the signal intensity of specific ions as a function of sample depth (represented by the sputter ion dose density), from the entire analysis area or from selected ROIs, (ii) ion images of selected ions at various sample depths, or (iii) mass spectra from specific 3D regions of the sample, defined by a selected 2D ROI and sputter ion dose density range.

The 3D ToF-SIMS analysis was done in a TOFSIMS 5 instrument (IONTOF GmbH, Germany) located at Linköping University, Sweden, using 30 keV Bi_3_^+^ primary ions for analysis and 10 keV Ar_2000_^+^ ions for sputtering. The beam currents were 0.18 pA and 5 nA for the Bi_3_^+^ and Ar_2000_^+^ ions, respectively. The sputter area was 500 × 500 µm^2^ and the analysis area was 200 × 200 µm^2^ to ensure homogenous dose densities within the analyzed area. The ratio of the analysis to sputter ion dose densities was 0.2%, in order to avoid accumulation of molecular damage caused by the Bi_3_^+^ ions [60]. The 3D ToF-SIMS data were acquired in the spectrometry analysis mode (bunched), whereas high-resolution ToF-SIMS images were recorded in the delayed extraction imaging mode (no bunching).

Data evaluation was done using SurfaceLab 7 (IONTOF GmbH, Münster, Germany) and Igor 9 (Wavemetrics, Inc., Lake Oswego, OR, USA). 

### 4.3. Scanning Electron Microscopy (SEM)

After 3D ToF-SIMS analysis, the sample was coated with a 15 nm film of Au/Pd and analyzed by SEM. SEM images were acquired in a Zeiss Supra 40VP microscope at an electron energy of 2 keV using an Everhardt-Thornley type detector (SE2).

## 5. Conclusions

3D ToF-SIMS analysis of corneocyte multilayers from human skin was used to map the spatial distributions of specific skin lipids in the layered SC structure. Whereas the dominating skin lipids, including long-chain fatty acids (C24:0 and C26:0), ceramides and cholesteryl sulfate, were relatively homogeneously distributed in the lipid matrix separating the corneocyte layers, a clearly distinct distribution was observed for cholesteryl oleate. The results demonstrate the capability of monitoring separately the molecular composition of individual corneocyte and lipid layers of SC, thus opening up the possibility to study penetration of exogenous compounds and the effect of penetration enhancers in unprecedented detail.

## Figures and Tables

**Table 1:** Peaks used to monitor different molecular components in SC during 3D ToF-SIMS analysis.

Negative Ions		
Observed *m*/*z*	Ion	Molecular assignment
50.005	C_3_N^−^	Proteins
60.020	C_2_H_4_O_2_^−^	Tape
66.001	C_3_NO^−^	Proteins
255.233	C_16_H_31_O_2_^−^	C16:0 fatty acid
281.248	C_18_H_33_O_2_^−^	C18:1 fatty acid
367.362	C_24_H_47_O_2_^−^	C24:0 fatty acid
395.392	C_26_H_51_O_2_^−^	C26:0 fatty acid
383.353	C_27_H_43_O^−^	Cholesterol
465.310	C_27_H_45_SO_4_^−^	Cholesteryl sulfate
708.666	C_44_H_86_NO_5_^−^	Ceramide AH (44:0)/AP (44:1)
738.708	C_46_H_92_NO_5_^−^	Ceramide AP (46:0)
**Positive Ions**		
Observed m/z	Ion	Molecular assignment
30.036	CH_4_N^+^	Proteins + ceramides
43.020	C_2_H_3_O^+^	Tape
57.078	C_4_H_9_^+^	Lipids
71.988	CNNa_2_^+^	Proteins
369.419	C_27_H_45_^+^	Cholesterol (esterified)
385.408	C_27_H_45_O^+^	Cholestrol (free)
650–820	C_x_H_y_NO_z_Na^+^	Ceramides (see Sjövall et al. [39] for specific assignments)
673.714	C_45_H_78_O_2_Na^+^	Cholesteryl oleate

## Data Availability

Data files will be provided upon request to the corresponding author.

## Supplementary Materials

The following supporting information can be downloaded at: https://www.mdpi.com/article/10.3390/ijms232213799/s1, Figure S1: SEM images of the edge of the corneocyte multilayer fragment; Figure S2: ToF-SIMS spectra of cholesterol and cholesteryl linoleate; Figure S3: ToF-SIMS images of single corneocyte layer after a sputter dose of 0.5 × 10^15^ cm^−2^.

## References

[1] Menon G.K., Cleary G.W., Lane M.E. (2012). The structure and function of the stratum corneum. Int. J. Pharm..

[2] Tobin D.J. (2006). Biochemistry of human skin—our brain on the outside. Chem. Soc. Rev..

[3] van Smeden J., Janssens M., Gooris G.S., Bouwstra J.A. (2014). The important role of stratum corneum lipids for the cutaneous barrier function. Biochim. Biophys. Acta.

[4] Boncheva M. (2014). The physical chemistry of the stratum corneum lipids. Int. J. Cosmet. Sci..

[5] Feingold K.R., Elias P.M. (2014). Role of lipids in the formation and maintenance of the cutaneous permeability barrier. Biochim. Biophys. Acta.

[6] Knox S., O’Boyle N.M. (2021). Skin lipids in health and disease: A review. Chem. Phys. Lipids.

[7] Moore D.J., Rawlings A.V. (2017). The chemistry, function and (patho)physiology of stratum corneum barrier ceramides. Int. J. Cosmet. Sci..

[8] Bouwstra J.A., Ponec M. (2006). The skin barrier in healthy and diseased state. Biochim. Biophys. Acta.

[9] Elias P.M., Williams M.L., Holleran W.M., Jiang Y.J., Schmuth M. (2008). Pathogenesis of permeability barrier abnormalities in the ichthyoses: Inherited disorders of lipid metabolism. J. Lipid Res..

[10] Elias P.M. (2012). Structure and function of the stratum corneum extracellular matrix. J. Investig. Dermatol..

[11] Iwai I., Han H., den Hollander L., Svensson S., Ofverstedt L.-G., Anwar J., Brewer J., Bloksgaard M., Laloeuf A., Nosek D. (2012). The human skin barrier is organized as stacked bilayers of fully extended ceramides with cholesterol molecules associated with the ceramide sphingoid moiety. J. Investig. Dermatol..

[12] Lundborg M., Wennberg C.L., Narangifard A., Lindahl E., Norlen L. (2018). Predicting drug permeability through skin using molecular dynamics simulation. J. Control. Release.

[13] Gregoire S., Luengo G.S., Hallegot P., Pena A.-M., Chen X., Bornschlögl T., Chan K.F., Pence I., Obeidy P., Feizpour A. (2020). Imaging and quantifying drug delivery in skin—Part 1: Autoradiography and mass spectrometry imaging. Adv. Drug Deliv. Rev..

[14] Pena A.M., Chen X., Pence I.J., Bornschlögl T., Jeong S., Gregoire S., Luengo G.S., Hallegot P., Obeidy P., Feizpour A. (2020). Imaging and quantifying drug delivery in skin—Part 2: Fluorescence andvibrational spectroscopic imaging methods. Adv. Drug Deliv. Rev..

[15] Hinder A., Schmelzer C.E., Rawlings A.V., Neubert R.H. (2011). Investigation of the molecular structure of the human stratum corneum ceramides [NP] and [EOS] by mass spectrometry. Skin Pharmacol. Physiol..

[16] Sadowski T., Klose C., Gerl M.J., Wójcik-Maciejewicz A., Herzog R., Simons K., Reich A., Surma M.A. (2017). Large-scale human skin lipidomics by quantitative, high-throughput shotgun mass spectrometry. Sci. Rep..

[17] van Smeden J., Hoppel L., van der Heijden R., Hankemeier T., Vreeken R.J., Bouwstra J.A. (2011). LC/MS analysis of stratum corneum lipids: Ceramide profiling and discovery. J. Lipid Res..

[18] Bouwstra J.A., Honeywell-Nguyen P.L., Gooris G.S., Ponec M. (2003). Structure of the skin barrier and its modulation by vesicular formulations. Prog. Lipid Res..

[19] Chen X., Gregoire S., Formanek F., Galey J.B., Rigneault H. (2015). Quantitative 3D molecular cutaneous absorption in human skin using label free nonlinear microscopy. J. Control. Release.

[20] Freudiger C.W., Min W., Saar B.G., Lu S., Holtom G.R., He C., Tsai J.C., Kang J.X., Xie X.S. (2008). Label-free biomedical imaging with high sensitivity by stimulated Raman scattering microscopy. Science.

[21] Mendelsohn R., Flach C.R., Moore D.J. (2006). Determination of molecular conformation and permeation in skin via IR spectroscopy, microscopy, and imaging. Biochim. Biophys. Acta.

[22] Chughtai K., Heeren R.M. (2010). Mass spectrometric imaging for biomedical tissue analysis. Chem. Rev..

[23] Rompp A., Spengler B. (2013). Mass spectrometry imaging with high resolution in mass and space. Histochem. Cell Biol..

[24] Bonnel D., Legouffe R., Eriksson A.H., Mortensen R.W., Pamelard F., Stauber J., Nielsen K.T. (2018). MALDI imaging facilitates new topical drug development process by determining quantitative skin distribution profiles. Anal. Bioanal. Chem..

[25] Hart P.J., Francese S., Claude E., Woodroofe M.N., Clench M.R. (2011). MALDI-MS imaging of lipids in ex vivo human skin. Anal. Bioanal. Chem..

[26] Gunnarsson A., Kollmer F., Sohn S., Hook F., Sjovall P. (2010). Spatial-resolution limits in mass spectrometry imaging of supported lipid bilayers and individual lipid vesicles. Anal. Chem..

[27] Massonnet P., Heeren R.M.A. (2019). A concise tutorial review of TOF-SIMS based molecular and cellular imaging. J. Anal. Atomic Spectrom..

[28] Winograd N. (2015). Imaging mass spectrometry on the nanoscale with cluster ion beams. Anal. Chem..

[29] Cizinauskas V., Elie N., Brunelle A., Briedis V. (2017). Skin Penetration Enhancement by Natural Oils for Dihydroquercetin Delivery. Molecules.

[30] Cizinauskas V., Elie N., Brunelle A., Briedis V. (2017). Fatty acids penetration into human skin ex vivo: ATOF-SIMS analysis approach. Biointerphases.

[31] Hagvall L., Pour M.D., Feng J., Karma M., Hedberg Y., Malmberg P. (2021). Skin permeation of nickel, cobalt and chromium salts in ex vivo human skin, visualized using mass spectrometry imaging. Toxicol. In Vitro.

[32] Holmes A.M., Scurr D.J., Heylings J.R., Wan K.W., Moss G.P. (2017). Dendrimer pre-treatment enhances the skin permeation of chlorhexidine digluconate: Characterisation by in vitro percutaneous absorption studies and Time-of-Flight Secondary Ion Mass Spectrometry. Eur. J. Pharm. Sci..

[33] Judd A.M., Scurr D.J., Heylings J.R., Wan K.W., Moss G.P. (2013). Distribution and Visualisation of Chlorhexidine Within the Skin Using ToF-SIMS: A Potential Platform for the Design of More Efficacious Skin Antiseptic Formulations. Pharm. Res..

[34] Kezutyte T., Desbenoit N., Brunelle A., Briedis V. (2013). Studying the penetration of fatty acids into human skin by ex vivo TOF-SIMS imaging. Biointerphases.

[35] Kubo A., Ishizaki I., Kubo A., Kawasaki H., Nagao K., Ohashi Y., Amagai M. (2013). The stratum corneum comprises three layers with distinct metal-ion barrier properties. Sci. Rep..

[36] Malmberg P., Guttenberg T., Ericson M.B., Hagvall L. (2018). Imaging mass spectrometry for novel insights into contact allergy—A proof-of-concept study on nickel. Contact Dermat..

[37] Okamoto M., Tanji N., Katayama Y., Okada J. (2006). TOF-SIMS investigation of the distribution of a cosmetic ingredient in the epidermis of the skin. Appl. Surf. Sci..

[38] Sjovall P., Greve T.M., Clausen S.K., Moller K., Eirefelt S., Johansson B., Nielson K.T. (2014). Imaging of distribution of topically applied drug molecules in mouse skin by combination of time-of-flight secondary ion mass spectrometry and scanning electron microscopy. Anal. Chem..

[39] Sjovall P., Skedung L., Gregoire S., Biganska O., Clement F., Luengo G.S. (2018). Imaging the distribution of skin lipids and topically applied compounds in human skin using mass spectrometry. Sci. Rep..

[40] Starr N.J., Hamid K.A., Wibawa J., Marlow I., Bell M., Pérez-García L., Barrett D.A., Scurr D.J. (2019). Enhanced vitamin C skin permeation from supramolecular hydrogels, illustrated using in situ ToF-SIMS 3D chemical profiling. Int. J. Pharm..

[41] Starr N.J., Johnson D.J., Wibawa J., Marlow I., Bell M., Barrett D.A., Scurr D.J. (2016). Age-Related Changes to Human Stratum Corneum Lipids Detected Using Time-of-Flight Secondary Ion Mass Spectrometry Following in Vivo Sampling. Anal. Chem..

[42] Bich C., Touboul D., Brunelle A. (2014). Cluster TOF-SIMS imaging as a tool for micrometric histology of lipids in tissue. Mass Spectrom. Rev..

[43] Fletcher J.S. (2015). Latest applications of 3D ToF-SIMS bio-imaging. Biointerphases.

[44] Shard A.G., Havelund R., Seah M.P., Spencer S.J., Gilmore I.S., Winograd N., Mao D., Miyayama T., Niehuis E., Rading D. (2012). Argon cluster ion beams for organic depth profiling: Results from a VAMAS interlaboratory study. Anal. Chem..

[45] Bich C., Havelund R., Moellers R., Touboul D., Kollmer F., Niehuis E., Gilmore I.S., Brunelle A. (2013). Argon cluster ion source evaluation on lipid standards and rat brain tissue samples. Anal. Chem..

[46] Henss A., Otto S.-K., Schaepe K. (2018). High resolution imaging and 3D analysis of Ag nanoparticles in cells with ToF-SIMS and delayed extraction. Biointerphases.

[47] Passarelli M.K., Newman C.F., Marshall P.S., West A., Gilmore I.S., Bunch J., Alexander M.R., Dollery C.T. (2015). Single-Cell Analysis: Visualizing Pharmaceutical and Metabolite Uptake in Cells with Label-Free 3D Mass Spectrometry Imaging. Anal. Chem..

[48] Starr N.J., Khan M.H., Edney M.K., Trindade G.F., Kern S., Pirkl A., Kleine-Boymann M., Elms C., O’Mahony M.M., Bell M. (2022). Elucidating the molecular landscape of the stratum corneum. Proc. Natl. Acad. Sci. USA.

[49] Sjovall P., Lausmaa J., Johansson B. (2004). Mass spectrometric imaging of lipids in brain tissue. Anal. Chem..

[50] Lehti S., Sjövall P., Käkelä R., Mäyränpää M.I., Kovanen P.T., Öörni K. (2015). Spatial distributions of lipids in atherosclerosis of human coronary arteries studied by time-of-flight secondary ion mass spectrometry. Am. J. Pathol..

[51] Okamoto M., Ishikawa K., Tanji N., Aoyagi S., Kita I., Migita C.T. (2012). Structural Analysis of the Outermost Hair Surface Using TOF-SIMS with C60 Depth Profiling Technique. E J. Surf. Sci. Nanotechnol..

[52] Coderch L., Lopez O., de la Maza A., Parra J.L. (2003). Ceramides and skin function. Am. J. Clin. Dermatol..

[53] Norlen L., Gil I.P., Simonsen A., Descouts P. (2007). Human stratum corneum lipid organization as observed by atomic force microscopy on Langmuir-Blodgett films. J. Struct. Biol..

[54] di Paolo G., Kim T.W. (2011). Linking lipids to Alzheimer’s disease: Cholesterol and beyond. Nat. Rev. Neurosci..

[55] Back M., Yurdagul A., Tabas I., Oorni K., Kovanen P.T. (2019). Inflammation and its resolution in atherosclerosis: Mediators and therapeutic opportunities. Nat. Rev. Cardiol..

[56] Brown M.S., Ho Y.K., Goldstein J.L. (1980). The cholesteryl ester cycle in macrophage foam cells. Continual hydrolysis and re-esterification of cytoplasmic cholesteryl esters. J. Biol. Chem..

[57] Ghosh S., Zhao B., Bie J., Song J. (2010). Macrophage cholesteryl ester mobilization and atherosclerosis. Vascul. Pharmacol..

[58] van der Molen R.G., Spies F., van t’Noordende J.M., Boelsma E., Mommaas A.M., Koerten H.K. (1997). Tape stripping of human stratum corneum yields cell layers that originate from various depths because of furrows in the skin. Arch. Dermatol. Res..

[59] Guo S., Domanov Y., Donovan M., Ducos B., Pomeau Y., Gourier C., Perez E. (2019). Anisotropic cellular forces support mechanical integrity of the Stratum Corneum barrier. J. Mech. Behav. Biomed. Mater..

[60] Havelund R., Seah M.P., Gilmore I.S. (2016). Sampling Depths, Depth Shifts, and Depth Resolutions for Bi(n)(+) Ion Analysis in Argon Gas Cluster Depth Profiles. J. Phys. Chem. B.

