# Supplementary material for: 3D Molecular Imaging of Stratum Corneum by Mass Spectrometry Suggests Distinct Distribution of Cholesteryl Esters Compared to Other Skin Lipids
Source: Int J Mol Sci. 2022 Nov 9;23(22):13799. doi: 10.3390/ijms232213799 (PMC9694581; doi:10.3390/ijms232213799)
Supplement: Supplementary file 1 [file ijms-23-13799-s001.zip › ijms-1924225-supplementary.pdf]

Supplementary Information for

3D molecular imaging of stratum corneum by mass spectrometry suggests distinct distribution of cholesteryl esters compared to other skin lipids

Peter Sjövall[1], Sebastien Gregoire[2], William Wargniez[2], Lisa Skedung[3], and Gustavo S. Luengo[2]

[1] RISE Research Institutes of Sweden, Materials and Production, SE-50115, Borås, Sweden
[2] L'Oréal Research and Innovation, 93601, Aulnay-sous-Bois, France
[3] RISE Research Institutes of Sweden, Bioeconomy and Health, SE-11428, Stockholm, Sweden

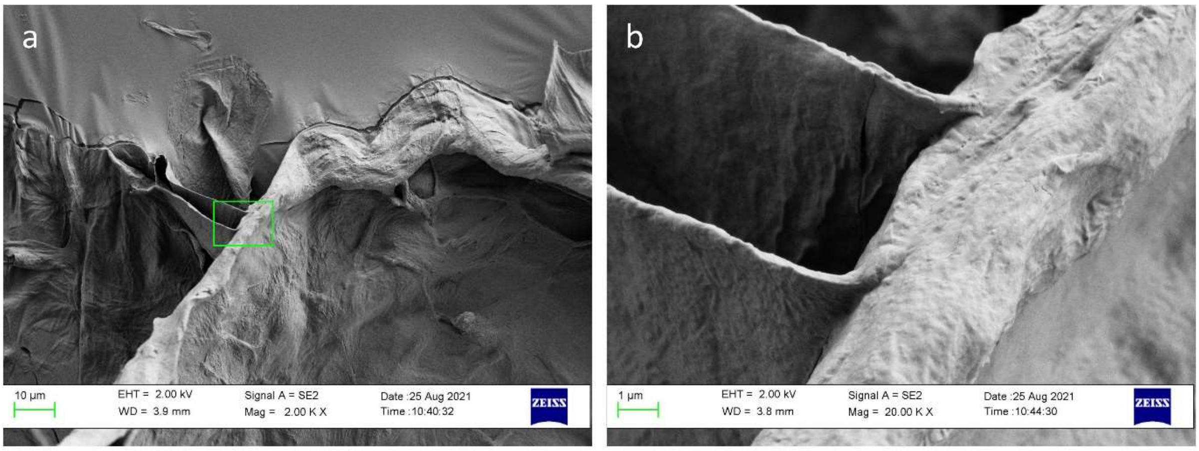

Supplementary Figure S1

SEM images of the edge of the corneocyte multilayer fragment analyzed by 3D-TOFSIMS. (b) is a magnified image of the area indicated by the green frame in (a). Note the single corneocyte layers partially removed from the skin fragment by attachment to the tape surface.

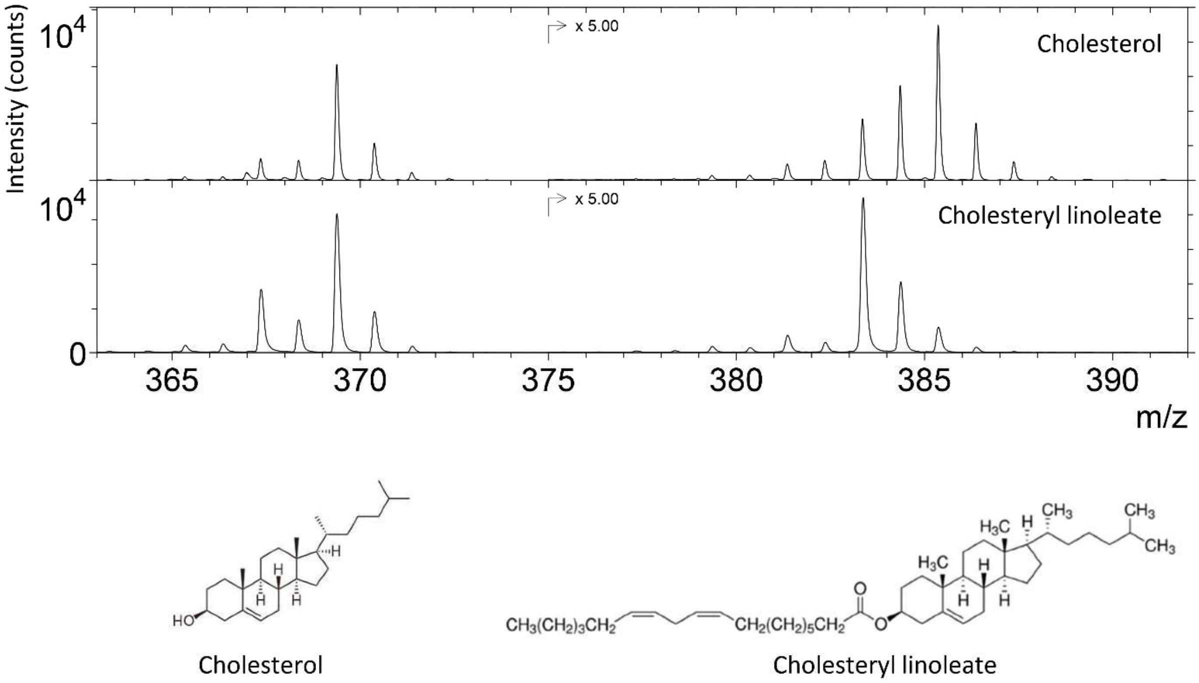

Supplementary Figure S2

Positive ToF-SIMS spectra of cholesterol and cholesteryl linoleate. The peaks at m/z 369.35 and 385.35 correspond to the cholesterol ions $C_{27}H_{45}^{+}$ and $C_{27}H_{45}O^{+}$, respectively.

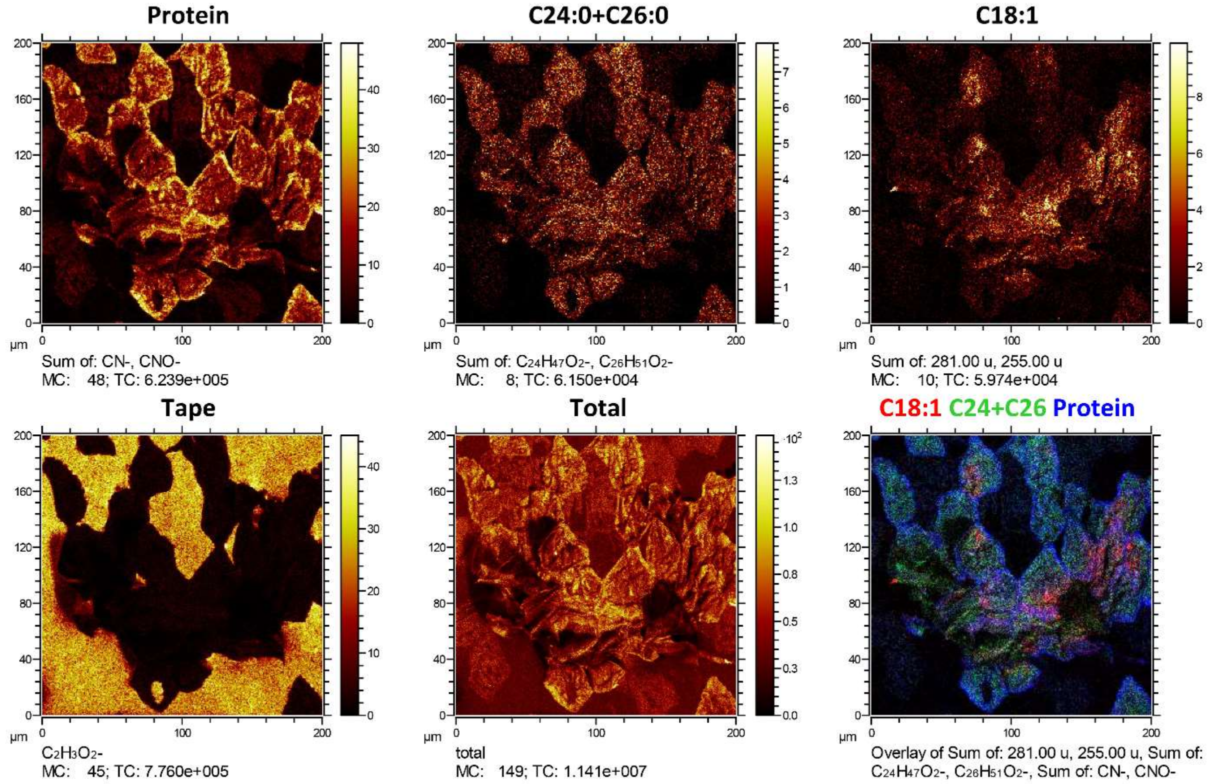

Supplementary Figure S3

Negative ion ToF-SIMS images of single corneocyte layer after a sputter dose of $0.5x10^{15}$ cm^{-2}. Single corneocytes are clearly distinguishable. Note the homogeneous distribution of C24:0+C26:0 fatty acids on the corneocyte surfaces and the considerably more inhomogeneous distribution of C16:0+C18:1.
